# Physiological Effects of Viewing Bonsai in Elderly Patients Undergoing Rehabilitation

**DOI:** 10.3390/ijerph15122635

**Published:** 2018-11-25

**Authors:** Chorong Song, Harumi Ikei, Masahiro Nara, Daisuke Takayama, Yoshifumi Miyazaki

**Affiliations:** 1 Center for Environment, Health and Field Sciences, Chiba University, 6-2-1 Kashiwa-no-ha, Kashiwa, Chiba 277-0882, Japan; crsong1028@chiba-u.jp (C.S.); ikei0224@ffpri.affrc.go.jp (H.I.); 2 Forestry and Forest Products Research Institute, 1 Matsunosato, Tsukuba, Ibaraki 305-8687, Japan; 3 Rehabilitation Center, Noda Hospital, 1554-1 Nakazato, Noda, Chiba 270-0237, Japan; naramasa17@yahoo.co.jp (M.N.); d1895cm@yahoo.co.jp (D.T.)

**Keywords:** older adults, nature therapy, heart rate variability, near-infrared spectroscopy, semantic differential method

## Abstract

The benefits of various nature-derived stimuli that can be used for stress relief and relaxation has recently gained immense attention; however, there are very few studies about their influence on elderly patients. The present study aims to present the effects of viewing bonsai on autonomic nervous activity, prefrontal cortex activity, and subjective assessment findings of psychological relaxation in elderly patients undergoing rehabilitation. Fourteen participants aged 64–91 years (mean age ± standard deviation, 78.6 ± 9.6 years) participated in this study. Miniature potted 10-year-old Japanese cypress bonsai trees were used as visual stimuli. Participants viewed the bonsai for 1 min, and the control comprised of no experimental stimulus. Physiological effects on autonomic nervous activity were assessed by measuring the heart rate variability (HRV) and pulse rate. The effects on prefrontal cortex activity were determined using near-infrared spectroscopy, which involved assessment of oxyhemoglobin concentrations in the left and right prefrontal cortices. Subjective evaluations were achieved by the modified semantic differential method. Viewing bonsai resulted in a significant increase in parasympathetic nervous activity, a significant decrease in sympathetic nervous activity, and a significant increase in the perceptions of feeling “comfortable” and “relaxed.” In conclusion, our findings indicated that viewing bonsai induces physiological and psychological relaxation.

## 1. Introduction

Along with the accelerated aging of the population, interest in enhancing the quality of life of the elderly is growing as well. It is clear that further research into the reduction of morbidity is necessary and that healthy living should be increasingly emphasized [1]. The primary focus of healthcare has been shifting from disease treatment to health promotion, disease prevention, and quality of life improvement. Recent studies have reported that natural environments, such as forests and urban parks, play an important role in health promotion and that nature-derived stimuli are positively associated with human health [2,3,4]. “Nature therapy” is defined as “a set of practices” aimed at achieving “preventive medical effects” through exposure to natural stimuli that render a state of physiological relaxation and boosts weakened immune functions to prevent diseases [2]. This therapy is increasingly recognized as an effective relaxation and stress management approach, and has the potential to be more widely adopted as an alternative and complementary therapy in the future.

As our modern lifestyle offers limited opportunities to get exposure to the natural environment, there is growing interest in the effects of nature-derived stimuli, which can be used daily in terms of stress relief and relaxation. A simple method to achieve contact with nature in an indoor setting is exposure to plants, such as foliage plants and fresh flowers, which are commonly used to decorate homes and offices. It is known that plants not only improve the quality of indoor air [5,6,7], but also help facilitate physiological and psychological relaxation through visual stimulation [2,8,9,10,11,12]. Previous studies have shown that viewing plants can decrease oxy-hemoglobin (oxy-Hb) concentration in the prefrontal cortex [8,9]; enhance parasympathetic nervous activity, which is increased in a relaxed state; suppress sympathetic nervous activity, which is increased in an aroused or stressed state; and decrease pulse rates [9,10,11]. Furthermore, viewing plants can increase positive feelings of comfort, relaxation, naturalness, and vigor, and decrease negative feelings of tension, anxiety, and fatigue [8,9,10,11]. A previous study examining the therapeutic effects of plants in a hospital environment found that systolic blood pressure and ratings of pain, anxiety, and fatigue were lower among patients in hospital rooms with plants than among those in rooms without plants [12].

However, the limitations that most previous studies faced were that they only investigated physiological responses associated with viewing plants in healthy young people. In a previous study, we demonstrated the physiological and psychological effects of viewing bonsai, which is a miniature natural landscape in a pot created using trees and other plants, in adult male patients with spinal cord injury [13]. We revealed that viewing bonsai significantly decreased left prefrontal cortex activity, increased parasympathetic nervous activity, decreased sympathetic nervous activity, increased positive feelings, and reduced negative feelings [13]. However, there have been no examinations on the physiological effects of viewing plants in elderly patients.

The aim of this study is to clarify the effects of viewing bonsai on autonomic nervous activity through measurements of heart rate variability (HRV) and pulse rate, prefrontal cortex activity through assessments using near-infrared spectroscopy (NIRS), and psychological relaxation through subjective assessments in elderly patients undergoing rehabilitation.

## 2. Materials and Methods

### 2.1. Participants

The study targeted Japanese elderly outpatients or hospitalized patients undergoing rehabilitation due to several conditions, such as lumbar compression fracture, femoral neck fracture, cerebral infarction, and cardiogenic cerebral embolism at Noda Hospital, Japan. The study included 14 patients (males, 4; females, 10) aged 64–91 years (mean age ± standard deviation, 78.6 ± 9.6 years). The height ranged from 139 to 175 cm (151.9 ± 10.1 cm) and weight ranged from 31 to 71 kg (51.4 ± 12.5 kg). Among the participants, patients with mild dementia were also included. All participants were informed about the aims and procedures of the study. After receiving a description of the experiment, they provided written consent to participate in the study. The study was conducted according to the Declaration of Helsinki, and the protocol was approved by the Ethics Committee of the Center for Environment, Health, and Field Sciences, Chiba University, Japan (project identification no.: 5).

### 2.2. Visual Stimulation

In this study, bonsai was used as visual stimuli. Bonsai has a characteristic of mimicking natural landscapes and is one of the nature-derived stimuli that has been used in daily life in Japan since a long time ago. In this experiment, miniature potted 10-year-old Japanese cypress bonsai trees modeling a forest landscape were used. Eight cypress trees, approximately 55 cm in height, were grouped together in a 40 × 20 × 5 cm ceramic pot. The distance from the participants’ eyes to the trees was 60–63 cm.

### 2.3. Experimental Design

Experiments were conducted in an experimental room at Noda Hospital, Japan. The room temperature, relative humidity, and illumination were 25.7 °C ± 0.7 °C, 66.3% ± 3.1%, and 383.0 ± 105.7 lx, respectively. After receiving a description of the purpose and outline of the study, the participants were moved into the experimental room while being seated on a wheelchair. Sensors for physiological measurement were fitted, and the participants received a detailed description about the experimental procedure. They then practiced the procedure using a foliage plant, once before the experiment.

**Figure 1:**
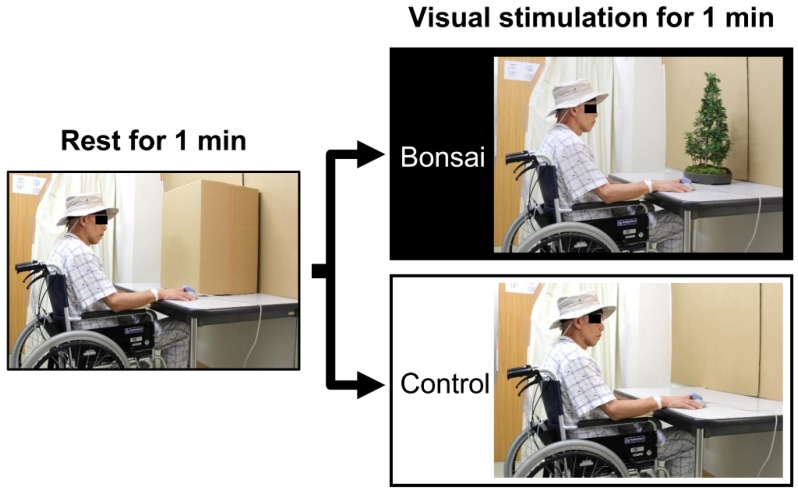
Experimental viewing conditions. Clockwise from left: rest condition, bonsai condition, and control condition.

The study protocol is presented in Figure 1. Before visual stimulation, the bonsai was covered with a corrugated cardboard box (rest condition; Figure 1 left). After a 1 min rest period, the participants viewed the bonsai (bonsai condition; Figure 1 upper right) or nothing (control condition; Figure 1 bottom right) for 1 min. All participants experienced both experimental conditions. During the testing procedure, the participants’ physiological responses were continually measured. After completion of the 1 min visual stimulation, subjective evaluations were conducted. To eliminate influences from the order of viewing the bonsai and the control, the visual stimuli were presented in a counterbalanced order.

### 2.4. Physiological Measurements

HRV and pulse rate were measured to assess autonomic nervous activity [14]. For assessment, the participants placed their forefingers on the sensor of an acceleration plethysmograph (APG; ARTETT, U-Medica Inc., Osaka, Japan). Previous studies have reported that the a–a intervals of an APG and the R–R intervals of an electrocardiograph are highly correlated [15]. Therefore, HRV was calculated by spectral analysis of the coefficient of variation of the a–a intervals of an APG. HRV was converted by a 60/a–a interval, and the sampling frequency was 1000 Hz. The power levels of the high frequency (HF; 0.15–0.40 Hz) and low frequency (LF; 0.04–0.15 Hz) components were calculated using the maximum entropy method (MemCalc/Win; GMS, Tokyo, Japan) [16]. HF power was considered to reflect parasympathetic nervous activity, and the LF-to-HF ratio (LF/HF) was considered to reflect sympathetic nervous activity [14,16]. To normalize the HRV parameters across participants, we used natural logarithmic-transformed values in the analysis [17]. In general, parasympathetic nervous activity is enhanced during relaxation, and sympathetic nervous activity is enhanced at the time of awakening or in situations of stress.

NIRS was used to assess brain activity [18,19]. The sensors were mounted at approximately Fp1 and Fp2 of the international 10–20 system (electroencephalogram) on each participant’s forehead. The oxy-Hb concentrations in the left and right prefrontal cortices were measured using a portable NIRS device (Pocket NIRS Duo, DynaSense, Hamamatsu, Japan). It is known that an increase or decrease in the quantity of blood flow in the brain is consistent with a corresponding increase or decrease in oxy-Hb [20], and it is thought that a decrease in the oxy-Hb concentration causes physiological calming.

### 2.5. Psychological Measurements

For the psychological measurements, the Japanese version of the modified semantic differential (SD) method was used [21]. The SD method subjectively assesses participants through a questionnaire with opposing adjectives, each of which is evaluated on a 13-point scale. Two pairs of adjectives were assessed as “comfortable–uncomfortable” and “relaxed–awakened.” Of the 14 participants, 9 had difficulties in filling out the questionnaire alone, and for these participants, hospital staff filled out the questionnaire for them after listening to their responses.

### 2.6. Data Analysis

All statistical analyses were performed using SPSS version 20.0 (IBM Corp., Armonk, NY, USA). Paired *t*-tests were used to compare physiological responses between before and after viewing bonsai (pre- vs. post-measurement) and between the two stimuli (bonsai vs. control). The Wilcoxon signed-rank test was used to compare psychological responses. Data are expressed as mean ± standard error (mean ± SE). For all analyses, a *p*-value < 0.05 was considered statistically significant. One-sided tests were used because we hypothesized that the participants would experience relaxation on viewing bonsai.

## 3. Results

**Figure 2:**
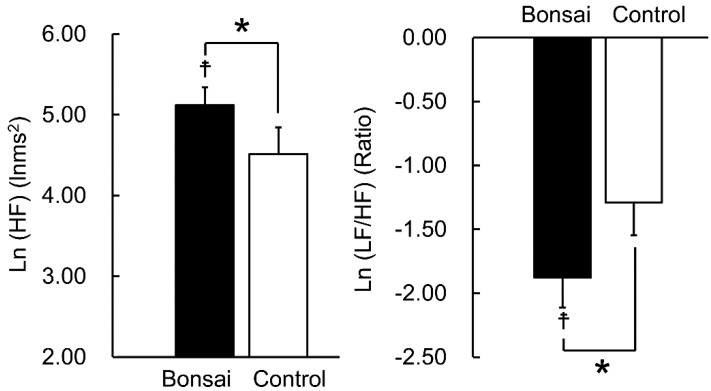
Heart rate variability during visual stimulation with bonsai vs. control. Left: parasympathetic nervous activity, mean natural logarithm (ln) of the high-frequency (HF) component; right: sympathetic nervous activity, mean natural logarithm (ln) of the ratio of low frequency (LF) to HF (LF/HF). Data are expressed as mean ± standard error (*n* = 12). ^‡^ *p* < 0.05 (before vs. after viewing), * *p* < 0.05 (bonsai vs. control), according to a paired *t*-test (one sided).

Figure 2 shows the comparison of the HRV results between viewing bonsai and the control. The mean ln(HF) values are presented in Figure 2 (left). On comparing the ln(HF) values before and after viewing bonsai, we found that the ln(HF) values were significantly higher after viewing bonsai than before viewing bonsai (5.12 ± 0.22 vs. 4.68 ± 0.37 lnms^2^; *p* = 0.043). Moreover, on comparing the two stimuli, we found that the ln(HF) values were significantly higher when viewing bonsai than when viewing the control (5.12 ± 0.22 vs. 4.51 ± 0.33 lnms^2^; *p*= 0.012). The mean ln(LF/HF) values are presented in Figure 2 (right). On comparing the ln(LF/HF) values before and after viewing bonsai, we found that the ln(LF/HF) values were significantly lower after viewing bonsai than before viewing bonsai (−1.88 ± 0.23 vs. −1.19 ± 0.26; *p* = 0.004). Furthermore, on comparing the two stimuli, we found that the ln(LF/HF) values were significantly lower when viewing bonsai than when viewing the control (−1.88 ± 0.23 vs. −1.29 ± 0.26; *p* = 0.048). However, there were no significant differences in the pulse rate on comparing between before and after viewing bonsai (69.2 ± 3.8 vs. 69.4 ± 3.8 beats/min; *p* = 0.326) and between viewing bonsai and the control (69.4 ± 3.8 vs. 69.0 ± 3.6 beats/min; *p* = 0.274). Moreover, there was no significant difference in respiratory frequency between viewing bonsai and the control (0.29 ± 0.06 vs. 0.30 ± 0.07 Hz; *p* = 0.188). Furthermore, the physiological responses were not significantly different between the rest condition with the cardboard box prior to viewing the bonsai and the control.

There were no significant differences in the changes in oxy-Hb concentrations in the left (bonsai: −0.14 ± 0.01 μM vs. control: −0.04 ± 0.01 μM; *p* = 0.219) and right prefrontal cortices (bonsai: −0.02 ± 0.01 μM vs. control: −0.04 ± 0.01 μM; *p* = 0.442) between viewing the bonsai and the control.

**Figure 3:**
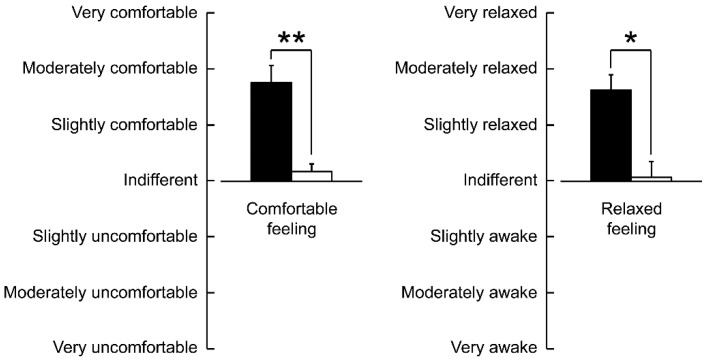
Subjective feelings as measured using a modified semantic differential questionnaire after visual stimulation with the bonsai vs. control. Data are expressed as mean ± standard error (*n* = 14). ** *p* < 0.01, * *p* < 0.05, according to the Wilcoxon signed-rank test (one sided).

Figure 3 shows the results of the modified SD method. Subjective evaluations indicated that the patients felt significantly more “comfortable (*p* = 0.004)” and “relaxed (*p* = 0.025)” when viewing the bonsai than when viewing the control.

## 4. Discussion

This study examined the physiological effects of viewing bonsai on autonomic nervous activity assessed using HRV and pulse rate, and on left and right prefrontal cortex activities assessed using NIRS. Furthermore, subjective assessments of psychological relaxation were conducted.

Results of physiological measurements showed that viewing bonsai significantly increased parasympathetic nervous activity and decreased sympathetic nervous activity. These results are partly consistent with the findings of previous studies on the effects of viewing foliage plants, fresh flowers, and bonsai [9,10,11,13]. However, there were no significant differences in pulse rate. A significant difference in HRV could be detected; however, no significant difference was detected in pulse rate because HRV is a more sensitive indicator than pulse rate. Previous studies on office workers to clarify the effects of viewing roses also showed a significant difference in HRV but not in pulse rate [11]. These results are partly consistent with the findings of the present study. Because parasympathetic nervous activity was induced, and sympathetic nervous activity was suppressed by viewing bonsai, it appears that viewing bonsai brings about physiological relaxation.

There were no significant differences in changes to oxy-Hb concentration in the left and right prefrontal cortices, and the reason for these insignificant differences in prefrontal cortex activity remains unknown. However, the oxy-Hb concentration did tend to decrease in the left prefrontal cortex when viewing the bonsai compared with the control, although the difference was not significant (bonsai: −0.14 ± 0.01 μM vs. control: −0.04 ± 0.01 μM; *p* > 0.05). It is necessary to obtain more data on the influence of plants on prefrontal cortex activity in future studies.

The results of subjective assessments of psychological relaxation showed that viewing bonsai significantly increased perceptions of feeling “comfortable” and “relaxed”, compared with the absence of bonsai.

This study showed that exposure to bonsai in an indoor environment could bring about effects of physiological and psychological relaxation in elderly patients undergoing rehabilitation. As elderly patients spend little time doing outdoor activities and spend more time in indoor environments compared to healthy young people, we believe that the finding of there being positive physiological and psychological relaxation effects with observation of indoor plants through the application of nature therapy is important. Furthermore, this finding may help promote the development of the environment, which is beneficial to the physical and mental health of elderly patients.

The present study had some limitations. The main limitation was the small sample size. To generalize the findings, further studies based on a larger sample including other demographic groups are required. Furthermore, a short stimulation duration of 1 min was used, and we do not know how long the effects will last. Future studies examining the duration of the effects following exposure to natural stimuli are required.

## 5. Conclusions

Our findings indicated that viewing bonsai induced physiological and psychological relaxation. Thus, nature therapy should be considered in elderly patients for improving quality of life.
